# Impact of distance education via mobile phone text messaging on knowledge, attitude, practice and self efficacy of patients with type 2 diabetes mellitus in Iran

**DOI:** 10.1186/2251-6581-11-10

**Published:** 2012-08-31

**Authors:** Mandana Goodarzi, Issa Ebrahimzadeh, Alireza Rabi, Bahman Saedipoor, Mohammad Asghari Jafarabadi

**Affiliations:** 1 Payame Noor University, Tehran, Iran; 2 Graduate School of Management, Farabi Institute of Higher Education, Karaj, Iran; 3 Biostatistics, Medical Education Research Center, Department of Statistics and Epidemiology, Faculty of Health and Nutrition, Tabriz University of Medical Sciences, Tabriz, Iran

**Keywords:** Distance education, Knowledge, Attitude, Practice, Self efficacy

## Abstract

**Objective:**

To evaluate the impacts of using SMS on improving laboratory test levels and Knowledge, Attitude, Practice (KAP) and Self Efficacy (SE) of patients with type 2 diabetes mellitus (DM) in Iran.

**Materials and methods:**

In this randomized controlled trial study, a total of 81 type 2 diabetes patients were randomly assigned into two groups exp. group (n = 43) and cont. group (n = 38). Only exp. group received 4 messages weekly consisted of diet, exercise, medication taking and. The researchers provided the intervention for 12 weeks. Data were collected with results of laboratory tests and KAP, SE reliable and valid questionnaires and demographic characteristics list. Data gathering at the baseline of the study and after 3 months intervention and was analyzed by SPSS11.5 software using descriptive and inferential statistics methods.

**Results:**

The results of this study showed that exp. group compared with cont. group improved significantly in HbA_1_C (p = 0.024), LDL (p = 0.019), cholesterol (p = 0.002), BUN (p ≤ 0.001), micro albumin (p ≤ 0.001), knowledge (p ≤ 0.001), practice (p ≤ 0.001) and self efficacy (p ≤ 0.001).

**Conclusion:**

The finding of this study demonstrate the effectiveness of intervention using SMS via mobile phone in the management of type 2 diabetes mellitus (DM). Thus, further studies are recommended for wide usage of distance education with mobile phone utilization.

## Introduction

The prevalence of type 2 (DM) in Iran has grown over the past decade [1]. Type 2 DM is a chronic and commonest disease requiring lifelong medical and life style adjustment [2]. Managing DM and its complications is very costly. Many studies have shown that control of hyperglycemia in diabetic patients can prevent or reduce the risks of diabetic complication [3]. Postprandial hyperglycemia has been associated with increased risk of micro- and macro vascular complications [4]. The glycemic control is poor among patients with diabetes in Iran like many other countries [5]. Better glycemic control can be achieved by improving patient’s knowledge, attitude, practice (KAP) and self efficacy (SE).

Study of Goodarzi et al. on type 2 DM patients of Karaj Diabetes Association (K.D.A) revealed middle knowledge, practice, SE and good attitude toward diabetes [6]. There is increasing amount of evidence that patient education is the most effective way to improve the patient’s KAP and SE [7]. Where traditional intervention have not been successful in reaching out to all, theory- based mobile e-health behavioral interventions are more likely to succeed and have the potential of lowering healthcare costs by lowering the use of healthcare resources. Cell phones, a common everyday technology, are already in the hands of millions of people. Harnessing this technology for improving the health of populations would be a step in the right direction [8].

New models of diabetes management systems using short message service (SMS) by mobile phone communication might be the most cost- effective tool for improving the quality of care for patients with type 2 diabetes. Mobile phones have had considerable impact in developing countries [9-11]. Communication by mobile phone is less expensive than alternative options such as land telephones or internet [12]. Three operators are currently serving more than 51 million mobile SIM cards in Iran, equating to penetration rate of mobile phone around 69%. SMS is widely used among mobile phone users especially the teenagers and adolescents. In early 2010, more than 40 million SMS’s were communicated each day by the main operator. This emerging telecommunication facility has provided new grounds of applications in health care. One of them is the investigated effectiveness of SMS education program on knowledge of patients about diabetes [3]. Another study investigated effects of SMS and telephone follow- up on improving HbA_1_C level [13]. Also, a study was done by Nesari et al. on follow- up over telephone to manage hyperglycemia and lipids for type 2 DM patients [14]. Except these studies yet, adequate attention has not been given to the use of this widely available and inexpensive tool in Iran. Hence, we decided in this study to investigate the effectiveness of an educational program via text massaging for management of type 2 DM patients from the Karaj, Iran. The aim is to evaluate how intervention through the SMS of mobile phone would improve blood glucose, lipid profile levels and other laboratory test, KAP and SE levels after 3 months period.

## Materials and methods

### Participants

In this randomized control trial study, participants were recruited from the Karaj Diabetes association (K.D.A.). The data were collected from 22 June to 22 September 2011 for a period of three months. Diabetic patients of both genders, above 30 years of age and ready to sign the consent form, were included in the study. Diabetes was diagnosed by specialist doctor according to the ADA criteria [15]. The inclusion criteria were diagnosed case of type 2 diabetes for more than one year, having mobile phone with ability to handle its SMS feature or having access to one belonging to a relative. The ability to read and write, sufficient vision power, having no problem in hearing and vocalization and no history of psychiatric diseases, HbA_1_C of above than 7%. They were excluded is if they had a clinical history of major illness such as renal insufficiency with creatinine level > 1.5 mg/dl, hepatic insufficiency and mental illness.

Sample size was determined by considering primary information on HbA_1_C from study by Zolfaghari, 2009 (SMS group: -1/01 (SD = 0/01) and telephone group: -0/03 (SD = 0/13) [13], and a confidence level of 95% and a power of 90%, utilizing Pocock formula at least 50 patients in each group. Finally 100 patients were selected on a random sampling scheme where in a list of random numbers was provided before data collection using the software. Admission order of the patients was considered as their ID number and based on the randomization on the ID number the patients were recruited in the study. For allocation to exp. and cont. groups, the researchers use RAS software and randomized by random permuted block design by a size of 2. From all patients in the exp. group (n = 50) and cont. group (n = 50) of the study population only 81 subjects completed the entire study, 43 subjects in exp. group and 38 subjects in cont. group with a response rate of 81% in total. The researchers excluded due to several reasons. In exp. group, two subjects admitted to hospital, one emigrated from Karaj, one bought a new mobile without Persian text message, three patients did not like to continue the study protocol. In control. group, five subjects were withdrawn from the study because they did not revisit the K.D.A. and seven subjects, did not completed questionnaires in post test Therefore, we report data from the 81 subjects who remained to complete the study protocol.

### Outcome measures

At enrollment, the patient^’^s demographic characteristics details, body mass index-BMI^a^ (kg/m^2^),family history of DM,^b^ information disease, disease complications, disease treatment, smoking status,^c^ systolic blood pressure(SBP) and diastolic blood pressure(DBP) were obtained in a suitably designed patient profile form. Additionally, laboratorial tests values like last first blood sugar or glucose (L-FBS), glycosylated hemoglobin (HbA_1_C), total cholesterol (TC), triglycerides (TG), high density lipoprotein cholesterol (HDL-C), low density lipoprotein cholesterol (LDL-C), micro albumin, BUN, creatinin (Cra) were recorded from the patient^’^s file, KAP and SE questionnaires were completed as pre - test data. The L-FBS, HbA_1_C, TC, TG, HDL-C, LDL-C, micro albumin, BUN, Cra, KAP and SE levels were measured again 3 months later (12 weeks) as post test data. Patient’s blood and urine were measured in the Bahar laboratory at the near of K.D.A. Patient^’^s blood was drown in vains for HbA_1_ C, FBS, lipids, Cr, BUN measurements. HbA_1_ was determined by a high-performance liquid chromatography technique.

### Measurement tools

#### Questionnaires

Prior to starting any education program it is appropriate to gauge the awareness level of the community under study by conducting a KAP study. This will help in implementing a health education program tailored to the needs of the particular community. A suitably designed and validated KAP and self efficacy questionnaire was administered at baseline and at the final follow-up to all the study patients to assess awareness regarding the disease and management. The questionnaires covered four areas: knowledge, attitude, practice and self efficacy. There were a total of 30 questions, consisting of 14 questions related to knowledge about diabetes, 5 questions to assess the attitude of the patient towards the disease, 6 questions regarding practices, which reflect how the patients put their knowledge and attitude into action and 5 questions about self efficacy. One point was awarded for each correct answer and none for an incorrect and unsure answer in part of knowledge and practice. Knowledge and practice (correct answer:1point, incorrect:0point). For part of attitude and self efficacy use Likert-scale in range 5 to 20 score. Attitude (completely agree:4point,agree:3point, disagree:2point, completely disagree:1point), self efficacy (completely sure:4point, fairly sure:3point, less sure:2point, have not sure absolotly:1point). These questionnaires were filled in a face to face interview with the investigator.

### Validity and reliability of the measures

The KAP and self efficacy showed good content validity (CVI values > 80% and CVR values > 99%) based on the evaluation of 6 expert in the field of internal medicine, endocrinology and metabolism disorders and education. The reliability of the measures were assessed based on both internal consistency and stability over time by repeating the questionnaire for a 30 randomly selected subjects. The measure showed reasonable internal consistency (α = 75%) and test- retest reliability (ICC = .80 and 95%, CI ≥ .9) [16,17].

### Suitably designed patient profile form

The patient’s demographic characteristics such as: sex (female: 1, male: 2), age (years), marital (single: 1, married: 2, widowed: 3), education level (reading and writing: 1, cycle: 2, diploma: 3, up diploma: 4, bsc and upper: 5), occupation (staff: 1, free: 2, unemployed: 3, house wife: 4, other: 5), child (number), physiological characteristic such as: family history (yes: 1, no: 2), body mass index (18.5-24.9: 1, 25.0-29.9: 2, 30-39.9: 3), smoking status (yes: 1, no: 2), length of time since diagnosis of diabetes or information disease (yes: 1, no: 2), disease complications (yes: 1, no: 2), disease treatment (without: 1, diet: 2, oral drugs: 3, insulin: 4, insulin & drugs: 5, insulin & diet: 6, oral drugs & diet: 7), Systolic blood pressure (mm/Hg) and Diastolic blood pressure (mm/Hg) and laboratory test values like: last fasting blood glucose (mg/dl), glycosylated hemoglobin (%), BMI (kg/m^2^), micro albumin μg/dl (yes: 1, no: 2), TC (mg/dl), HDL-C (mg/dl), LDL-C (mg/dl), TG (mg/dl) BUN (mg/dl), Cr (mg/dl) were recorded from the patient’s exam report were obtained in a suitably designed patient profile form. Changes in the KAP and SE score, glycemic control and lipid profile were investigated at the end of study. The KAP and SE questionnaires responses were analyzed and used to develop an appropriate educational program.

## Intervention

The goal of the intervention was to maintain blood glucose, lipids, BUN, creatinin, within a normal range and HbA_1_C < 7% with increase of patient’s knowledge, attitude, practice and self efficacy about diabetes. Before the intervention, each patient was instructed by a researcher about how to use their own mobile phone and to check their ability to read SMS and match the time for sending message and telephone follow- up. The researchers provided the intervention for 3 months (12 weeks). Patients in the exp. group could receive 4 messages weekly consisting of information about exercise, diet, diabetic medication, important of self- monitoring blood glucose levels and they could receive the messages at any place where access was possible by mobile phone. Recommendations covered four areas, included knowledge, attitude, practice and self efficacy. Sample recommendations related to knowledge were as follows:

"*”the symptoms of hypoglycemia are hungry, pail, chilling, sweating cold, confusion, palpitation”, ”every 6–12 months visit by ophthalmologist”.* Related to attitude were *:”diabetes disease can be control if you change your life style”,”a significant of patient are success in daily life”.* Related to practice were: *”please increase vegetables in every meal”, ”please examine your feet every day”*. Related to self efficacy were: *”try to visit by your family physician every two months”, ”self monitoring of your blood glucose is necessary”*."

Overall 48 messages were sent to patients during the intervention. The control group did not receive any educational message during this time by researchers.

For ethical considerations, the research protocol was approved by ethics committee of Islamic Azad University- Karaj and this trial is registered with Iranian Registery of Clinical Trials. Registration ID in IRCT is IRCT201112158416N1.

Written consent was obtained from these patients who agree to participate in the study. Anonymity and confidentiality were guaranteed to participants.

## Statistical analysis

All analyses were performed using SPSS 11.5 software (SPSS Inc, Chicago IL) the data were summarized by mean (SD) and frequency (percent) for quantitative and qualitative variables respectively. The normality of the numeric variables was tested and confirmed using K-S test. The comparison of proportions defined by categorical variables were compared using chi square tests between experimental and control groups. Independent samples t-tests were used to investigate the differences of numeric variables between experimental and control groups before intervention and for assessing these differences after intervention Analysis of Covariance (ANCOVA) has been used adjusting for baseline measurements and confounders(such as age and information disease). For qualitative variable the Mantel-Hansel test was used to assess after intervention differences adjusting for baseline measurements. The differences between before and after intervention were tested using paired samples T-tests and Mc-Namara Tests in each group respectively for quantitative and qualitative variables. P < 0.05 was considered as significant.

## Results

The mean age of exp. group was 50.98 (SD = 10.32) years and that of the control group was 56.71 (SD = 9.77) years. The mean of information disease was 6 years in exp. group and 10 years in control group. There was no significant difference in sex, marital, education, occupation, family history, smoking status, disease complications and disease treatment. At the pre- test, no significant difference was found in BMI, last FBS, HbA_1_C, LDL, TG, CT, BUN, Cr, BP, micro albumin, knowledge, practice and self efficacy levels between the groups (Table 1). There was a significant change in HbA_1_C (p = 0.024), LDL (p = 0.19), cholesterol (p = 0.002), BUN (p ≤ 0/001), micro albumin (p < 0.001) for the exp. group (Table 2). There was a significant change in knowledge (p < 0.001), practice (p < 0.001) and SE (p < 0.001) for the exp. group (Table 3). For other characteristics there was no differ significantly.

**Table 1:** Baseline demographic and clinical data of the examination and control groups

**Characteristics**	**Exp (n=43)**	**Cont (n=38)**
Age (years)*	50.98(SD= 10.32)	56.71(SD= 9.77)
**Sex**		
female	34(79.1%)	29(76.3%)
male	9(20.9%)	9(23.7%)
**Marital**		
single	1(2.3%)	0(0%)
married	41(95.3%)	37(97.4%)
widowed	1(2.3%)	1 (2.6%)
**Education**		
Reading and Writing	13(30.2%)	22(57.9%)
Cycle	11(25.6%)	5(13.2%)
Diploma	12(27.9%)	10(26.3)
Up Diploma	4(9.3%)	0(0%)
BSC and Upper	3(7%)	1(2.6%)
**Occupation**		
Staff	5(11.6%)	4(10.5%)
Non-government	6(14%)	5(13.2%)
Unemployed	1(2.3%)	0(0%)
Housewife	30(69.8%)	26(68.4%)
Other	1(2.3%)	3(7.9%)
Number of children**	Median: 3.0	Median: 4.0
	percentiles: 2.0 – 5.0	percentiles: 3.0 – 5.0
**family history**		
Yes	31(75.6%)	30(78.9%)
No	10(24.4%)	8(21.1%)
**BMI (**kg /m^2^ )		
18.51	7(16.3%)	10(26.3%)
25/25.01	23(53.5%)	22(57.9%)
30/30.01+	13(30.2%)	6(15.8%)
**Smoking**		
Yes	6(14%)	3(7.9%)
No	37(86%)	35(92.1%)
**Inform. Disease** (years)***	Median: 6.0	Median:10.0
	percentiles: 2.0 – 15.0	percentiles: 6.0 – 15.0
**Disease Complication**		
Yes	18(41/9%)	20(52.6%)
No	25(58/1%)	18(47.4%)
**Disease Treat**		
Without	1(2.3%)	0(0%)
Diet	1(2.3%)	1(2.6%)
Oral Drugs	20(46.5%)	19(50%)
Insulin	10(23.3%)	14(36.8%)
Insulin and Oral Drugs	7(16.3%)	1(2.6%)
Insulin and Diet	1(2.3%)	0(0%)
Oral Drugs and Diet	3(7.9%)	3(7.9%)
**Micro albumin (μg/dl)**		
Yes	21(48.8%)	24(63.2%)
No	22(51.2%)	14(36.8%)
**BMI (kg/m**^**2**^**)**	28.51(SD=3.90)	27.37(SD=3.49)
**SBP (mm/Hg)**	127.91(SD=15.20)	126.84(SD=16.45)
**DBP (mm/Hg)**	74.65(SD=12.21)	78.68(SD=9.34)
**LFBS (mg/dl)**	161.49(SD=54.15)	151.47(SD=55.59)
**HbA**_**1**_**C (%)**	7.91(SD=7.24)	7.83(SD=1.12)
HDL-C (mg/dl)****	43.67(SD=10.75)	49.08(SD=12.45)
**LDL-C (mg/dl)**	43.67(SD=10.75)	99.13(SD=28.78)
**TG (mg/dl)**	179.72(SD=99.83)	173.42(SD=87.00)
**TC (mg/dl)**	180.88(SD=41.47)	176.92(SD=31.15)
**BUN (mg/dl)**	24.58(SD=7.67)	24.76(SD=8.57)
**Cr (mg/dl)**	0.9395(SD=0.18534)	0.8974(SD=0.21746)
**Knowledge**	7.97(SD=2.58)	8.05(SD=2.11)
Attitude *****	18.25(SD=4.32)	16.73(SD=1.91)
**Practice**	3.72(SD=1.18)	3.86(SD=0.77)
**SE**	15.34(SD=2.6 4)	15.86(SD=2.74)

* = P<0.05Data are: mean (SD) / n (%).

* p = 0.12, ** p = 0.12, *** P = 0.27.

**** p= 0.39, ***** p= 0.49.

**Table 2:** Comparison of the results laboratory tests of pre- and post- test in exp. and control groups

**Group**	**Exp**		**Cont**		
**Characteristics**	**Before**	**After**	**Before**	**After**	**P**
**L- FBS**	161.49(SD = 54.15)	133.56(SD = 36.44)	151.47(SD = 55.59)	142(SD = 38.00)	0.234
**HbA1C**	7.91(SD = 1.24 )	7.02(SD = 1.02)	7.83(SD = 1.12)	7.48(SD = 1.26)	0.24 ^a^
**HDL**	43.67(SD = 10.75)	43.72(SD = 9.20)	49.08(SD = 12.45)	46.26(SD = 10.17)	0.448
**LDL**	97.88(SD = 36.26)	87.93(SD = 29.95)	99.13(SD = 28.78)	98.95(SD = 29.64)	0.19 ^a^
**TG**	179.72(SD = 99.83)	160.16(SD = 71.930	173.42(SD = 87.0)	169.08(SD = 66.89	0.372
**TC**	180.88(SD = 41.47)	165.95(SD = 38.18)	176.92(SD = 31.15)	187.29(SD = 38.67	0.002 ^a^
**BUN**	24.58(SD = 7.67)	20.37(SD = 5.190)	24.76(SD = 8.57)	24.11(SD = 7.41)	<0.001 *^a^
**Cr**	0.9395(SD =0.18534	0.881(SD = 0.1803)	0.8974(SD = 0.21746)	0.911(SD = 0.1886)	0.097
Micro Albumin # @^b^	9(42.9%)	12(57.1%)	2(8.3%)	22(91.7%)	<0.001 *^a^

$: P-value based on ANCOVA.

# : P-value based on Mantel-Hansel test.

*: P<0/05.

Mean (SD) is reported unless for Micro Albumin where N (%) is reported.

@: No significant differences were observed based on McNamara Tests in experimental and control group.

^**a**^ = p < 0/05.

N (%).

^**b**^ p = 0.000.

**Table 3:** Comparison of the results of KAP and SE questionnaires of pre- and post- test in exp. and control groups

**Group**	**Exp**		**Cont**			
**Characteristics**	**Before**	**After**	**Before**	**After**	**F(1.77)**	**P**
Knowledge	7.97(SD = 2.58)	10.83(SD = 2.15)	8.05(SD = 2.11)	8.68(SD = 1.97)	45.24	<0.001 *^a^
Attitude	18.25(SD = 4.32)	18.16(SD = 1.25)	16.73(SD = 1.91)	17.15(SD = 1.77)	1.59	0.211
practice	3.72(SD = 1.18)	4.93(SD = 1.16)	3.86(SD = 0.77)	4.26(SD = 0.92)	23.88	<0.001 *^a^
SE	15.34(SD = 2.64)	17.02(SD = 1.150	15.86(SD = 2.74)	15.31(SD = 2.67)	41.19	<0.001 *^a^

^**a**^ = P < 0/05.

There was a significant percentage change in knowledge (p < 0/001), with a mean change of 53.95 (7.97 pre- test to 10.83 after three months), and also in practice for the exp. group (p < 0/001), with a mean change of 38.57 (3.72 pre-test to 4.93 after three months) and percentage change in SE (p < 0/000), with a mean change of 13.19 (15.34 pre-test to 17.02 after three months (Table 4).

**Table 4:** Results of mean (SD = Std. Deviation) of percent change

**Group**	**Exp**	**Cont**
**Characteristics**	**Mean (SD=std )**	**Mean (SD=std)**
Knowledge	53.95(SD=80.02)	10.29(SD=18.96)
Attitude	1.67(SD=10.15)	2.87(SD=7.43)
practice	38.57(SD=36.59 )	10.43(SD=13.98 )
SE	13.19(SD=16.24)	-3.10(SD=7.80)

## Discussion

Management of D.M. not only requires the prescription of appropriate nutritional and pharmacological regime by the physician but also intensive education of the patient [18]. Large studies about this relationship have used characteristics such as HbA_1_C, lipids, KAP and SE as the index of diabetes management [3,13,19,20]. This study revealed that text message via mobile phone intervention could improve KAP, SE and laboratory test levels in patients with type 2 diabetes in a randomized control trial. In this study, HbA_1_C levels decreased in exp. group greater than cont. group after 3 months compared with two baseline (p = 0/24). Previous studies showed the following result: a comparative study to effectiveness of SMS versus telephone call on HbA_1_C in type 2 diabetes resulted in a decrease level of HbA_1_C 1/01% in SMS group and 0/93% in telephone group after 12 weeks [13]. Results of this study showed that LDL (p = 0/19) and total cholesterol (p = 0/002) levels decreased in exp. Group after 3 months compared with cont. group; but other lipids such as TG and HDL did not differ significantly with two groups. In a similar study, Kim and Yoon reported that, total cholesterol, TG and HDL did not differ significantly [20]. Nesari et al. reported that 12 weeks follow- up examination of HbA_1_C and lipids levels in D.M. patients by telephone call management caused mean decreased of HbA_1_C, TG, total cholesterol and LDL; but similar our study, in HDL level did not differ significant [14]. It is believed that the period of intervention was not enough to change the HDL levels. In this study BUN (p ≤ 0/001) and micro albumin (p ≤ 0/001) decreased in exp. group compared with cont. group. Measurement of these items did not show in any study. This study shown that sending educational SMS via mobile phone is significantly effective in increasing knowledge of exp. group compared cont. group (p ≤ 0/001). Fatehi et al. reported that educated via SMS for 45 days could increase knowledge level in exp. group compared cont. group. They suggested that this intervention period was short and changing in attitude and SE must be regarded for further studies [3]. Ferrer- Roca et al. and Roberto E. Isquierdo et al. in similar study achieved same result [21,22].

In our study practice (p ≤ 0.001) and SE (p ≤ 0.001) levels was increased in exp. group after 3 months compared with cont. group; but attitude level did not differ significantly with two groups. It is believed that change in the attitude level needs a long term period. The higher percentage of yes answers from patients with positive family history of diabetes was surprising, (exp. group = 75.6%, cont. group = 78.9%). A study by Javadi et al. [23] showed that patients with positive family history were more aware of the role of heredity, of diet as a mode of therapy, and of long- term complications of diabetes mellitus. This shows the rapid emergence of diabetes mellitus among the general population. We also observed that 83.7% of exp. group and 75.9% of cont. group were overweight or obese. A study by Malathy et al. [19] showed similar result, which indicates the stressful life style and the poor level of awareness regarding the benefits of physical activity and exercises in reducing the BMI. About the strength of this study, it can be pointed out that the outcomes such as KAP, SE and metabolic control of patients are comprehensive which is not the case in similar studies. The limitation of this study was that 19 subjects of participants were lost and it seems related to role of motivation in self care. Also the intervention period was not enough to change the variables such as attitude and HDL.

## Conclusion

It seems that having better understanding of the effectiveness of mobile phone intervention in improving healthcare results and its process; larger controlled and possibly multicenter studies with longer period in intervention need to be used. Healthcare providers should be willing to use this common technology. Thus, studies are needed for effectiveness of costs and technical financial studies and adaption with real clinical situations. Mobile phones, with the combination of voices and text messages, their independent and flexible situations are a great opportunity for designing and providing the healthcare interventions for the community.

## Endnotes

^a^BMI: The weight (in kilograms) divided by the square of the height (in meters). Classification of overweight and obesity were as per the recommendations of the national heart, lung. And blood institute.1998.according to this classification. patients with BMI of 18.5-24.9 kg/m^2^ were considered as normal,25.0-29.9 kg/m^2^ was over weight and 30.0-39.9 kg/m^2^ was considered obese.

^b^ Family history of DM: Diabetic patients whose first-degree relatives developed DM before the age of 60.were categorized as patients with a positive history of DM.

^c^ Smokers: Individuals who reported to have smoked 100 cigarettes in their lifetime, irrespective of whether they were current smokers or not.

## Competing interests

The authors declare that they have no competing interests.

## Authors’ contributions

MG contributed to the conception and design of this study and drafting the manuscript. IEZ contributed to the conception and design of this study. AR and BS contributed to the revised the manuscript critically for important intellectual content. MAJ contributed to the analysis and interpretation of data and revising the article critically for important intellectual content. All authors read and approved the fianl manuscript.
